# Primary chemoradiation with cisplatin versus cetuximab for locally advanced head and neck cancer: a retrospective cohort study

**DOI:** 10.1186/s40164-020-00175-1

**Published:** 2020-08-05

**Authors:** Il Seok Daniel Jeong, Huan Mo, Anthony Nguyen, Esther G. Chong, Hsin Hsiang Clarence Tsai, Justin Moyers, Matthew Kim, Curtis Lacy, Vivek Shah, Eric Lau, Yi Xu, Huynh Cao

**Affiliations:** 1 School of Medicine, Loma Linda University, Loma Linda, CA USA; 2 Department of Pathology, Loma Linda University Medical Center, Loma Linda, CA USA; 3 Department of Hematology and Oncology, Loma Linda University Medical Center, 11175 Campus Street, CSP 11015, Loma Linda, CA 92354 USA; 4 Department of Medicine, Loma Linda University Medical Center, Loma Linda, CA USA; 5 Department of Regenerative Medicine, Loma Linda University Medical Center, Loma Linda, CA USA

**Keywords:** Head and neck squamous cell carcinoma, Cisplatin, Cetuximab, Chemoradiation therapy, HPV/p16

## Abstract

**Objective:**

To explore the efficacy of primary chemoradiation with cisplatin versus cetuximab with respect to HPV/p16 and smoking statuses.

**Methods:**

We retrospectively reviewed patients from our center with locally advanced non-nasopharyngeal head and neck squamous cell carcinoma (HNSCC) who received primary chemoradiation with cisplatin or cetuximab between 2006 and 2018.

**Results:**

The median OS for cisplatin (n = 66) was not reached versus 132 months when treated with cetuximab (n = 55) (p = 0.03). For HPV/p16-positive patients, we found the median OS for cisplatin (n = 34) was not reached versus 60 months with cetuximab (n = 21) (p = 0.036). In the smoking group, the median OS was not reached in the cisplatin group (n = 44) versus 60 months when treated with cetuximab (n = 32) (p = 0.03).

**Conclusion:**

HPV/p16-positive and smoking cohorts treated with cisplatin-based chemoradiotherapy had a significantly better OS versus cetuximab.

## Background

Head and neck squamous cell carcinoma (HNSCC) is the sixth most common cancer in the world, with 650,000 new cases diagnosed each year. Tobacco use, heavy alcohol intake, and carcinogenic human papillomavirus (HPV) are strong risk factors [1, 2]. HPV-positive HNSCC responds favorably to chemoradiation leading to improved prognosis [3, 4].

Patients who have locoregionally advanced disease at diagnosis receive combined modality therapy with a platinum-based chemotherapy backbone; [5, 6] however, in 2006, cetuximab, a chimeric monoclonal antibody and an epidermal growth factor receptor (EGFR) inhibitor, was approved with radiation therapy for locoregionally advanced HNSCC. The addition of cetuximab to radiotherapy improved survival in HNSCC [7]. Cetuximab has been proposed as an alternative to platinum chemotherapy due to a more favorable toxicity profile and improved tolerability in those with renal disease or in the elderly. However, two recent multi-centers randomized clinical trials in patients with HPV associated HNSCC have demonstrated both inferior overall survival of patients who received cetuximab based instead of cisplatin-based radiotherapy and no difference in terms of toxicity profile [8, 9]. One hypothesis for the decreased efficacy of cetuximab in HPV associated HNSCC is the decreased dependence on the EGFR pathway compared to HPV-negative tumors [10].

We aimed to compare real-world outcomes of cisplatin-based versus cetuximab-based primary chemoradiation in an academic setting at Loma Linda University Medical Center. We also re-analyzed the HNSC dataset from The Cancer Genome Atlas (TCGA) [11] to clarify the relationship between HPV and *EGFR* statuses in HNSCC.

## Materials and methods

### Data collection

ICD-9, ICD-10, and CPT codes were used to search the Loma Linda University Health Cancer Registry to find patients diagnosed with HNSCC between 2006 and 2018. This study was approved by Loma Linda University Medical Center Institutional Review Board (IRB Approval #: 5180298). The following data were obtained for each patient: age, sex, smoking status, primary malignancy site, pathology report, staging, type of radiation and number of radiation treatments, chemotherapy regimen, and number of chemotherapy cycles completed. Radiation dose ranged between 48 and 72 Gy.

### Patient selection

Eligible patients were between the ages of 18–99 years who had locally advanced head and neck cancer with initial stages III-IVB receiving concurrent chemoradiation with either cisplatin or cetuximab as first-line therapy. Primary tumors of the oral cavity, oropharynx, and hypopharynx/larynx were included. We excluded those who underwent surgery as first-line therapy, concurrent chemoradiation as second-line or salvage therapy for recurrent or metastatic disease, underlying malignancy other than head and neck cancer, those who did not receive either cisplatin or cetuximab as part of chemotherapy regimen, and nasopharyngeal tumors.

Cases that met inclusion criteria were then separated by chemotherapy received. Categorical variables analyzed included smoking and HPV/p16 statuses, which was abstracted from pathology reports consistent with College of American Pathologists recommendation [12]. p16^INK4a^ immunohistochemistry stain and HPV in situ hybridization were performed in Clinical Laboratory Improvement Amendments regulated laboratories with in vitro diagnostic antibodies. Patients with a smoking history were further categorized into light smoking (< 10 pack-years) and heavy smoking (≥ 10 pack-years). Comorbidities was considered positive if patients had ≥ 2 comorbidities and negative if < 2.

### Endpoints and statistics

Primary endpoint was overall survival (OS) by treatment with either cisplatin or cetuximab. OS was analyzed by the Kaplan–Meier and log-rank methods. Survival was compared by univariate Cox proportional hazards model between treatment groups. Survival analyses were stratified by HPV/p16 and smoking statuses. Statistical analysis with p-values of < 0.05 was considered significant. Statistics were performed with R packages “survival” and “survminer” (Vienna, Austria).

### TCGA data and analysis

Publicly available data for clinical and phenotypic information, gene expression (by RNA sequencing), and copy number (generated by the Affymetrix Genome-Wide Human SNP Array 6.0 platform) data of the HNSC cohort from TCGA were obtained from University of California Santa Cruz Xena Browser. Gene expression data were represented as RSEM scores, which were log2-transformed normalized counts [13]. The copy number estimated values are thresholded to −2, −1, 0, 1, 2, representing homozygous deletion, single copy deletion, diploid normal copy, low-level copy number amplification, or high-level copy number amplification reported by GISTIC2 [14, 15]. *EGFR* copy number statuses were grouped to amplified (GISTIC2 score ≥ 1) and non-amplified (GISTIC2 score ≤ 0). HPV status was obtained from the supplementary information of the original Nature article [11], and most of them were ascertained by the presence of HPV transcripts or genomes by whole genome/exome and RNA sequencing. All analyses were performed on KNIME Analytics.

## Results

### Patient and tumor characteristics

**Fig. 1:**
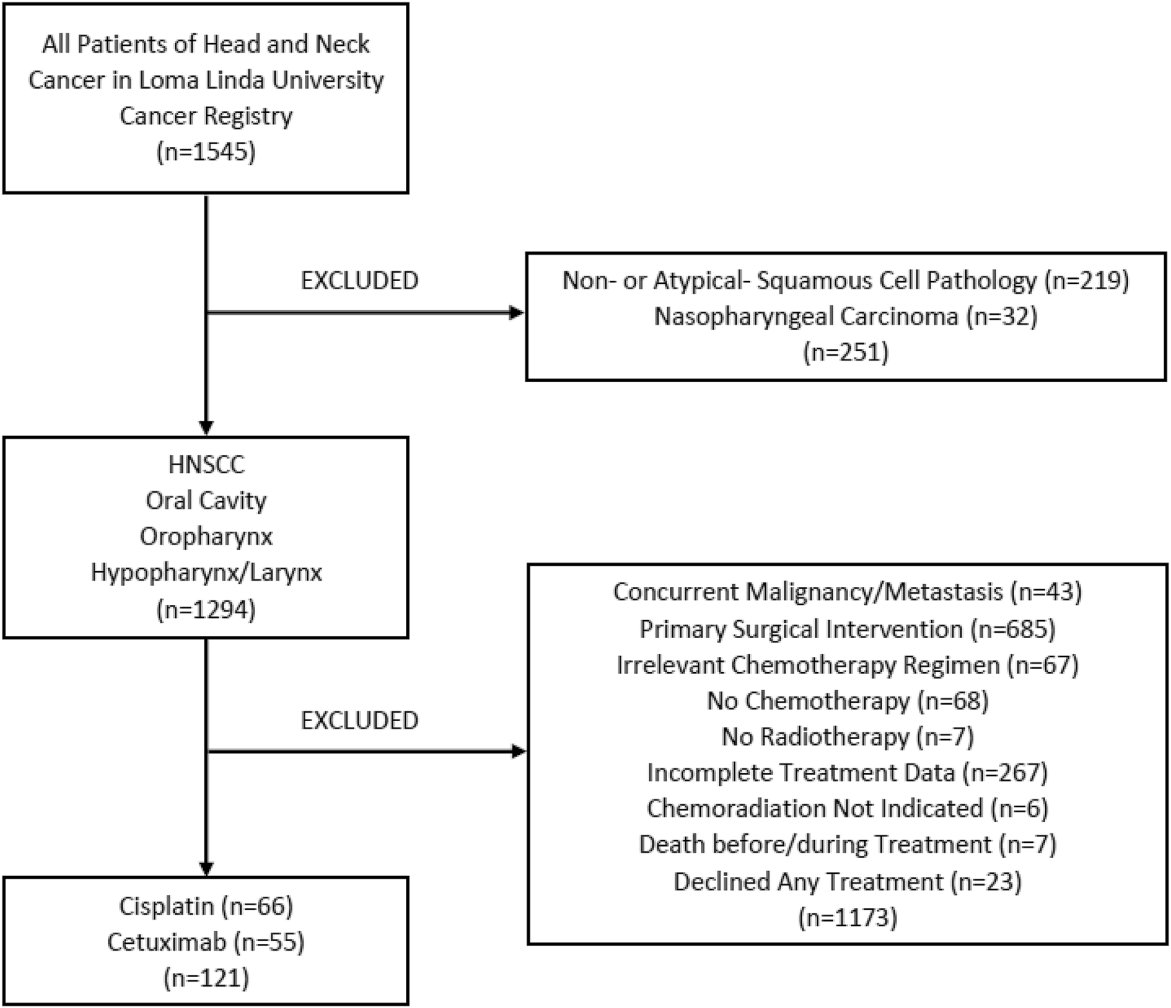
Patient screening flowchart

A total of 1545 patients were retrieved from the LLUMC Cancer Registry between 2006 and 2018. Of these patients, 121 met inclusion criteria and were further divided into cisplatin (n = 66) and cetuximab (n = 55) cohorts (Fig. 1). A summary of demographic information can be found in Table 1.

**Table 1:** Patient characteristics based on primary chemotherapy received

Parameters	Cisplatin (n = 66)	Cetuximab (n = 55)
Sex		
Male	51	39
Female	15	16
Age		
> 60	18	33
≤ 60	48	22
Mean ± SD (Overall 60.09 ± 10.28)	56.80 ± 9.10	60.04 ± 10.30
HPV/p16		
Positive	34	21
Negative	9	7
Not available	23	27
Primary		
Oropharynx	55	45
Hypopharynx/Larynx	5	7
Oral Cavity	5	2
Other	1	1
Elevated baseline creatinine (> 1.3 mg/dL)		
Yes	14	13
No	43	38
Not Available	9	4
Smoking		
Smoker	44	32
Light (< 10 Pack-Years)	10	4
Heavy (≥ 10 Pack-Years)	26	25
Unknown Pack-Years	8	3
Non-Smoker	21	21
Not Available	1	2
Comorbidities		
Yes (≥ 2 Conditions)	48	35
No (< 2 Conditions)	16	16
Not Available	2	4
T Staging		
T1	6	11
T2	26	20
T3	20	14
T4	12	9
Tx	2	1
N staging		
N0	3	7
N1	8	8
N2	46	36
N3	7	2
Nx	2	2
M staging		
M0	60	51
M1	0	0
Mx	6	4

### Cisplatin chemoradiation improves OS

**Fig. 2:**
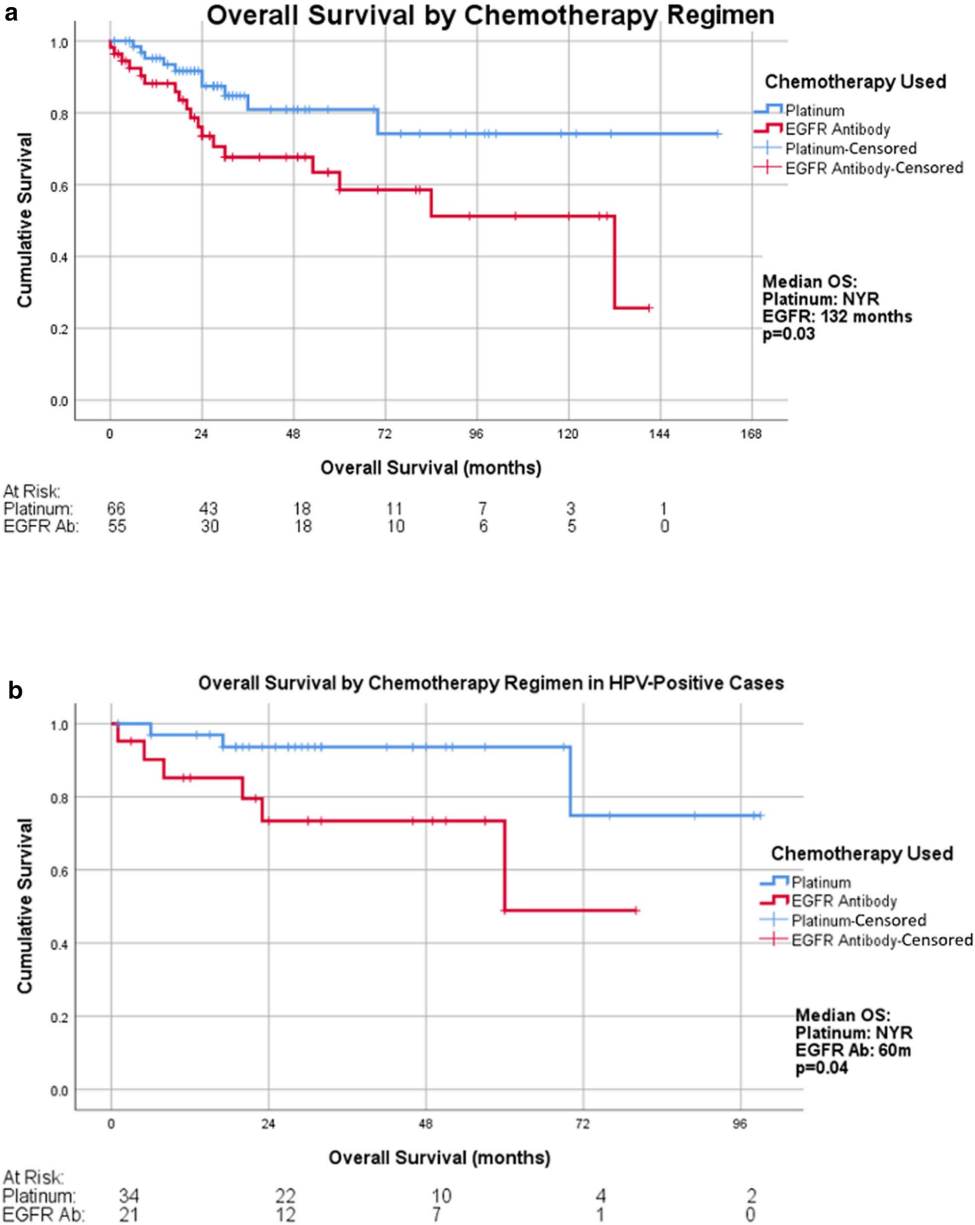

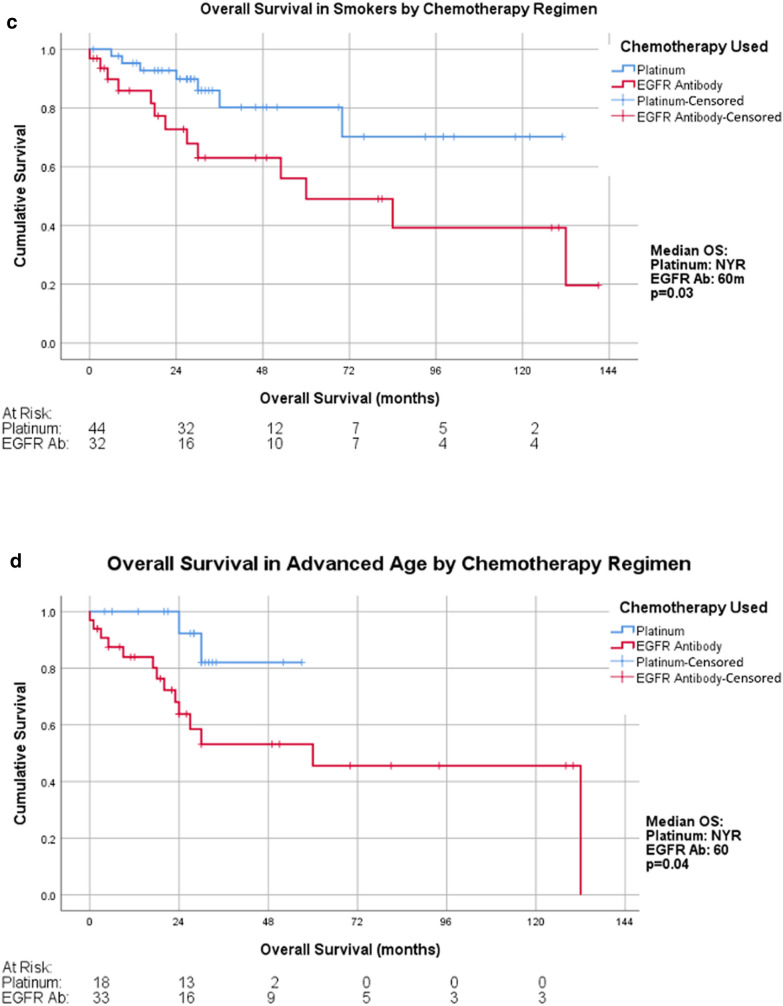
Overall survival of (**a**) the overall study population, (**b**) the HPV/p16-positive population, (**c**) the smoking population, and (**d**) the advanced age population that received primary chemoradiation with cisplatin versus cetuximab

The median OS for patients receiving cisplatin was not reached compared to 132 months in the cetuximab group (p = 0.03, Hazard Ratio [HR] = 0.44, [95% confidence interval (CI), 0.20–0.95]) (Fig. 2a).

### Improved OS in HPV/p16-positive cancer treated with cisplatin-based chemoradiation

Among the patients who were HPV/p16-positive (n = 55), those who received cisplatin chemoradiation had longer OS. The median OS for patients with HPV/p16-positive cancer treated with cisplatin-based therapy was not reached compared to 60 months for HPV/p16-positive cancer treated with cetuximab (p = 0.036, HR = 0.25 [95% CI 0.061–1.00]) (Fig. 2b).

### Cisplatin chemoradiation improved OS in patients with positive smoking history

In patients with a smoking history (n = 76), cisplatin-based radiotherapy was associated with longer OS. The median OS in the cisplatin group was not reached, while that in the cetuximab cohort was 60 months (p = 0.03, HR = 0.36 [95% CI 0.14−0.93]) (Fig. 2c).

### Age and outcomes

Within our study population, patients in the cisplatin group were significantly younger than those in the cetuximab group (56.80 versus 60.04, respectively, p = 0.0003). Of patients over 60 years old (n = 51), those treated with cisplatin had improved OS (p = 0.035, HR = 0.23, [95% CI 0.051−1.03]) (Fig. 2d).

### TCGA HNSC data re-analysis

**Fig. 3:**
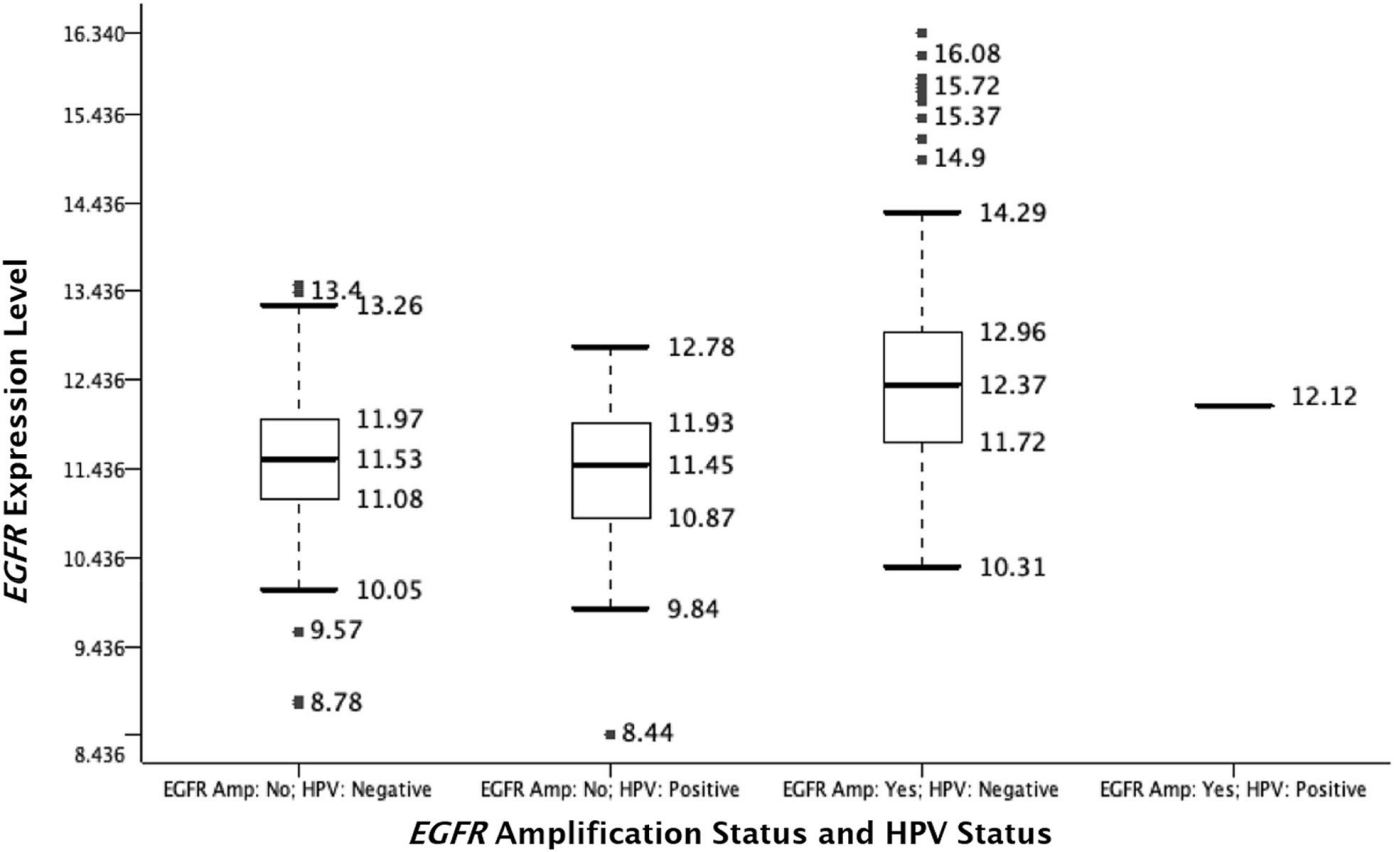
Comparisons of *EGFR* expression levels of HNSCC cases in TCGA HNSC cohort based on HPV status and amplification (Amp) status of the *EGFR* gene region (x-axis). The *EGFR* expression level (y-axis) by RNA-sequencing is presented in RSEM scores [13] on a log2 scale

Within TCGA who had HPV-positive cancers, none of the oropharyngeal (n = 22) and only one of the overall head and neck patients (n = 36) had the amplified *EGFR* gene region, whereas within the HPV-negative cohort, six oropharyngeal (n = 11, 54.5%) and 124 of the overall head and neck patients (n = 243, 51.0%) had the amplified *EGFR* gene region. Fisher’s exact tests indicated that the HPV-negative cohort is more likely to possess *EGFR* amplification in head and neck patients (p < 0.0001). At the transcription level, RNA expressions of the *EGFR* gene in HPV-negative cancers were 1.619 times (95% CI 1.22 to 2.14) higher than HPV-positive cancers in the overall head and neck cohort (p < 0.0001) (Fig. 3).

## Discussion

In this single institution retrospective study, we investigated real-world outcomes of locoregionally advanced HNSCC treated with primary chemoradiation with cisplatin versus cetuximab.

Our finding of an OS benefit in HPV/p16-positive cases treated with cisplatin-based therapy compared to cetuximab is consistent with recent phase 3 clinical trial data [8, 9] and national database studies [16–18]. While our study and the VA study by Bauml et al. both found that cetuximab with radiation yields inferior OS, more than 99% of the VA study population are male and it did not perform a subgroup analysis of HPV status. This growing evidence supports cisplatin-based radiotherapy as the standard of care for patients with HPV/p16-positive cancer with low risk (e.g. non-smokers or < 10 pack-years smoking history) [9]. We also found the survival benefit with cisplatin therapy to be consistent among those who smoke and those with advanced age. However, cetuximab likely still will play a role in patients with poor renal function, as both even though head to head trials suggested no different overall toxicity, cisplatin consistently demonstrated increased rates of acute kidney injury [8, 9].

Some authors have proposed that the inferior survival outcomes in patients with HPV associated oropharyngeal HNSCC who received concurrent chemoradiation with cetuximab instead of cisplatin might be related to differences in EGFR expression and HPV status. Indeed, we observed that amplification of the *EGFR* gene region was rare to absent in HPV-positive cancers, while about half of the HPV-negative cases possessed different degrees of amplifications in the TCGA HNSC dataset. Expression of the *EGFR* gene was also significantly lower in HPV-positive HNSCC, consistent with prior reports [19]. However, EGFR expression has not been shown to be predictive of clinical benefit for anti EGFR therapy in SCCHN, which may be related to the lack of correlation between EGFR expression and autophosphorylation activity [20, 21].

The main limitation of this study is that, as a retrospective study, we cannot exclude possible confounding factors that may contribute to inferior OS of cetuximab-based radiotherapy in patients with HPV/p16-positive cancers, such as insufficient renal function that have precluded a cisplatin regimen or the selection bias of cetuximab in elderly and frail patients. However, based on our data even the population with advanced age still benefited from high dose cisplatin, and further more patients with at least 2 comorbidities received cisplatin treatment. In conclusion, our result was consistent with the recent prospective randomized clinical trials, which have provided strong evidence of the superiority of cisplatin compared with cetuximab in HPV/p16-positive cancers [8, 9].

## Data Availability

The single institute data from this study is available from the corresponding author upon reasonable request.

## Abbreviations

HNSCC: Head and neck squamous cell carcinoma
TCGA: The Cancer Genome Atlas
OS: Overall survival
EGFR: Epidermal growth factor receptor
HPV: Human papillomavirus

## References

[1] Falk RT (1989). Effect of smoking and alcohol consumption on laryngeal cancer risk in coastal Texas. Cancer Res.

[2] Vokes EE (1993). Head and neck cancer. N Engl J Med.

[3] Vigneswaran N, Williams MD (2014). Epidemiologic trends in head and neck cancer and aids in diagnosis. Oral Maxillofacial Surg Clinics North Am.

[4] Marur S (2010). HPV-associated head and neck cancer: a virus-related cancer epidemic. Lancet Oncol.

[5] Bachaud JM (1996). Combined postoperative radiotherapy and Weekly Cisplatin infusion for locally advanced head and neck carcinoma: Final report of a randomized trial. Int J Radiation Oncol Biol Phy.

[6] Szturz P, Vermorken JB (2016). Treatment of elderly patients with squamous cell carcinoma of the head and neck. Front Oncol.

[7] Rosenthal DI (2015). Association of human papillomavirus and p16 status with outcomes in the IMCL-9815 phase III registration trial for patients with locoregionally advanced oropharyngeal squamous cell carcinoma of the head and neck treated with radiotherapy with or without cetuximab. J Clin Oncol.

[8] Gillison ML (2019). Radiotherapy plus cetuximab or cisplatin in human papillomavirus-positive oropharyngeal cancer (NRG Oncology RTOG 1016): a randomised, multicentre, non-inferiority trial. Lancet.

[9] Mehanna H (2019). Radiotherapy plus cisplatin or cetuximab in low-risk human papillomavirus-positive oropharyngeal cancer (De-ESCALaTE HPV): an open-label randomised controlled phase 3 trial. Lancet.

[10] Oosthuizen JC, Doody J (2019). De-intensified treatment in human papillomavirus-positive oropharyngeal cancer. Lancet.

[11] Lawrence MS (2015). Comprehensive genomic characterization of head and neck squamous cell carcinomas. Nature.

[12] Lewis JS (2017). Human papillomavirus testing in head and neck carcinomas: guideline from the College of American Pathologists. Arch Pathol Lab Med.

[13] Li B, Dewey CN (2011). RSEM: accurate transcript quantification from RNA-Seq data with or without a reference genome. BMC Bioinform.

[14] Mermel CH (2011). GISTIC2.0 facilitates sensitive and confident localization of the targets of focal somatic copy-number alteration in human cancers. Genome Biol.

[15] Zack TI (2013). Pan-cancer patterns of somatic copy number alteration. Nat Genet.

[16] Bauml JM (2019). Cisplatin versus cetuximab with definitive concurrent radiotherapy for head and neck squamous cell carcinoma: an analysis of Veterans Health Affairs data. Cancer.

[17] Riaz N (2016). Concurrent chemoradiotherapy with cisplatin versus cetuximab for squamous cell carcinoma of the head and neck. Am J Clin Oncol.

[18] Tang C (2015). Concurrent cetuximab versus platinum-based chemoradiation for the definitive treatment of locoregionally advanced head and neck cancer. Head Neck.

[19] Lassen P, Overgaard J, Eriksen JG (2013). Expression of EGFR and HPV-associated p16 in oropharyngeal carcinoma: correlation and influence on prognosis after radiotherapy in the randomized DAHANCA 5 and 7 trials. Radiother Oncol.

[20] Kriegs M (2019). Analyzing expression and phosphorylation of the EGF receptor in HNSCC. Sci Rep.

[21] Bossi P (2016). Prognostic and predictive value of EGFR in head and neck squamous cell carcinoma. Oncotarget.

